# Angiotensin II in vasodilatory shock: lights and shadows

**DOI:** 10.1186/s13054-017-1869-9

**Published:** 2017-11-14

**Authors:** Elio Antonucci, Sara Agosta, Yasser Sakr

**Affiliations:** 1 Intermediate Care Unit, Guglielmo da Saliceto Hospital, Piacenza, Italy; 2 Department of Anesthesia and Intensive Care, Uniklinikum, Jena, Germany

Data from the literature show lights and shadows about the use of angiotensin II (Ang II), for instance as an alternative vasopressor in patients with vasodilatory shock that requires high doses of catecholamines. Recently, an international randomized controlled trial (ATHOS-3) [1] has shown that Ang II can induce a significant increase in mean arterial pressure (MAP) if compared to placebo. Moreover, during the first 48 hours from the randomization, doses of the vasopressors (norepinephrine (NE) and vasopressin) were significantly reduced in the Ang II group but not in the placebo group. Interestingly, no difference in adverse effects was remarkable between the two groups.

However, some important issues need to be clarified before any definitive conclusion about Ang II in vasodilatory shock. Firstly, we do not know exactly the timing for Ang II initiation: is it better to add Ang II only when NE doses jump to 0.2 μg/kg/min or when NE requirements rapidly increase (e.g., 0.5 μg/kg every hour)? Secondly, Ang II could be administered to specific patients. In previous studies, some patients were extremely sensitive to Ang II infusion (e.g., medication with ACE inhibitors; sartans or beta-blockers) [2, 3]. Furthermore, cirrhotic patients usually show a reduced angiotensinogen synthesis with secondary low circulating levels of Ang II [4]. In this perspective, could we hypothesize that an early infusion of Ang II has a positive effect on these patients? Thirdly, the safety profile of Ang II has never been tested in patients with vasodilatory shock and concurrent myocardial dysfunction. According to the case of the nonselective nitric oxide synthase inhibitor [5], Ang II could reduce the cardiac output due to its preferential vasoconstrictive action and provide some detrimental effects for those patients with myocardial dysfunction. Finally, Ang II significantly increased the heart rate (HR) in the ATHOS-3 trial. However, Ang II should not have a positive chronotropic effect and the authors did not manage to provide us with a reason for this phenomenon. We can only hypothesize that the increased HR is related to a relative hypovolemia. However, also in this case no clear information about the volemic status was found in the ATHOS-3 trial (e.g., total fluid administration or total fluid balance; cardiac index measurements missed in 56% of cases).

In conclusion, Ang II is doubtless a promising vasopressor but some questions still need to be answered before any definitive conclusion in the field.

## Abbreviations

Ang II: Angiotensin II
HR: Heart rate
MAP: Mean arterial pressure
NE: Norepinephrine
